# Mixed effects logistic regression analysis of blood pressure among Ghanaians and associated risk factors

**DOI:** 10.1038/s41598-023-34478-0

**Published:** 2023-05-12

**Authors:** Abdul-Karim Iddrisu, Ishmael Besing Karadaar, Joseph Gurah Junior, Bismark Ansu, Damoah-Asante Ernest

**Affiliations:** 1 Department of Mathematics and Statistics, University of Energy and Natural Resources, Sunyani, Ghana; 2 Department of Mathematics and ICT, St. Ambrose College of Education, Dormaa-Akwamu, Ghana; 3 Department Mathematics and Science, Nkoranman Senior High School, Seikwa, Ghana

**Keywords:** Hypertension, Statistics, Epidemiology

## Abstract

Blood pressure (BP) control is a global health issue with an increase in BP beyond the normal BP leading to different stages of hypertension in humans and hence the need to identify risk factors of BP for efficient and effective control. Multiple BP measurement have proven to provide BP readings close to the true BP status of the individual. In this study, we used multiple BP measurement data on 3809 Ghanaians to determine risk factors associated with BP. The data were obtained from World Health Organization study on Global AGEing and Adult Health. We defined high blood pressure (HBP) as $\ge$ 130/80 mmHg or normal as $\le$ 130/80 mmHg. We provide summary statistics and also used the Chi-Square test to assess significance of association between HBP versus risk factors of HBP. The aim of this study is to identify risk factors of BP using the mixed effects logistic regression model. Data were analyzed using R version 4.2.2. The results showed that the risk of high blood pressure (HBP) decreases across the three measurement periods. There is reduced risk (OR = 0.274, 95% CI = 0.2008, 0.405) of HBP among male participants relative to female participants. The risk (OR = 2.771, 95% CI = 1.8658, 4.1145) of HBP increased by 2.771-folds among those who are 60 years and above relative to those below the age of 60 years. Those whose work involves/requires vigorous exercise has 1.631-fold increase in the risk (OR = 1.631, 95% CI = 1.1151, 2.3854) of HBP relative to those whose work does not involve vigorous exercise. There is approximately 5-folds increased in the risk (OR = 4.896, 95% CI = 1.9535, 12.2268) of among those who have ever been diagnosed with diabetes. The results also revealed high risk (OR = 1.649, 95%CI = 1.1108, 2.4486) of HBP among those who have formal education. The risk (OR = 1.009, 95% CI = 1.0044, 1.0137) of HBP increases with increasing weight and a reduced risk (OR = 0.996, 95% CI = 0.9921, 0.9993) of HBP with increasing height. We found that sad experience, either mild, moderate or severe, is associated with a reduced risk of HBP. Those who have vegetable servings at least 2 cups per day have increased risk of HBP and those who have fruits servings at least 2 cups per day is associated with a reduced risk of HBP, however this is not statistically significant. To achieve success in BP control, programs should be designed with the aim of reducing weight, educate those with formal eduction on issues relating to HBP. Those whose work requires vigorous exercise are recommended to have regular check-ups to ensure that pressure build-up in the lungs is cleared. SBP is lower for women at young age but continue to increase after menopause as their BP increase becomes salt-sensitive. Hence there is need to give more attention to menopausal women so as to improve BP. Both young and old individuals are recommended to practice regular exercise since this has shown to reduce risk of being overweight or becoming diabetic and reduces the risk of HBP at yong age and old age. Also, to improve blood pressure control, programs for management of blood pressure or hypertension should focus more short stature individuals since such people are more likely to experience HBP.

## Introduction

High Blood pressure is a global health problem, especially for developing countries^1,2^. Hypertension, which is associated with high BP (HBP) has become an increasingly common public health problem. Hypertension is known to be the most common cause of death globally^3–5^. The Joint National Committee on Prevention, Detection, Evaluation, and Treatment of HBP (JNC7) defined hypertension as a SBP $\ge$140 mmHg and DBP $\ge 90$ mmHg^6^. Other reports/guidelines also accept these value as a tool for hypertension diagnosis^6–8^. Hypertension arises when the blood pushing against the walls of arteries in the body creates pressure higher than the normal BP^9,10^. The SBP and DBP are often used to measure BP. The SBP is the pressure in the arteries when the heart beats, whereas the DBP is the pressure in the arteries when the heart rests. For normal BP, the SBP is less than 120 millimetres of mercury (mmHg), and the DBP is less than 80 mmHg, often described/written as 120/80 mmHg. Every country has an adopted definition for high blood pressure which is more often guided by the WHO criteria. Some guidelines suggest that HBP should be defined as BP which is consistently higher than 130/80 mmHg, while WHO guidelines suggest higher than 140/90 mmHg^9–11^. Elevated SBP is a leading global health risk^12,13^. Various studies^12–15^ have shown that SBP of at least 110 mmHg is associated with multiple cardiovascular and renal outcomes, including ischemic heart disease, cerebrovascular disease, and chronic kidney disease. Global obesity epidemic is likely to increase SBP among certain group of people^3,12,13^.

The global policy agenda aimed at controlling BP remains a challenge, and BP-related hypertension is the leading cause of cardiovascular disease (CVD) worldwide^16^. In Africa, the prevalence of hypertension is the highest relative to rest of the world with 27% of adults having hypertension^16^. Hypertension is a major public health issue in Sub-Saharan Africa^17^ where it is estimated that about 10-20 million people are living with hypertension^18^. The 2014 Ghana Health Service report indicates that high number of out-patient cases is attributed to hypertension. There is consistent increase in hypertension prevalence in Ghana^19^. Studies have shown that BP-related hypertension is one of the leading cause of admission and mortality in Ghana^19–21^. In 2017, hypertension accounted for 4.7% of the total admissions and 15.3% of total deaths in Ghana^21^. Hypertension is the leading cause of medical emergencies such as heart^22^ and renal^23^ failures and it is the key risk factor of stroke in Ghana. The incidence of stroke among people who are above 60 years increases with increasing level of BP^24^. Also, the Ghanaian Society of Cardiology (GSC), the Stroke Association Support Network-Ghana (SASNET-Ghana) in partnership with the World Heart Federation, reported that an estimated 34% of the population between the ages of 30 and 70 years has hypertension or high blood pressure. These bodies revealed that this percentage is slightly more than half of the estimated number of people living with hypertension^25^.

Literature on BP revealed that the burden of SBP still remains high even though there are preventive interventions and anti-hypertensive medications that are effective and low-cost^12,13^. The paradigm of determining high BP or hypertension has shifted towards SBP^26^. For instance Lloyd-Jones and colleagues^27^ revealed that SBP is more likely to correctly classify subjects as having high BP or hypertension relative to diastolic BP. The article by Pogue et al.^28^, published in 1996, also noted that it is SBP, not the DBP, that is the key determinant of appropriate classification of middle-aged and older persons as well as those undergoing treatment as having high BP or hypertension. Using the data from the Framingham Heart Study cohort, Kannel et al.^29^ found in 1971 that SBP is an appropriate determinant of outcomes such as high BP, hypertension and cardiovascular diseases. Several other studies confirmed this finding including that of Ali and colleagues^30^. It took 22 years for SBP to be recognized as even equally important to DBP in classifying individuals as having high BP or hypertensive^26,27^. Lloyd-Jones et al.^27^ pointed out the importance of SBP measurement and Fisher^31^ even suggested that we should ignore DBP measurement. But for the fact that pulse rate is a better predictor of events than SBP, especially in older persons^32–34^ and we cannot compute pulse rate without DBP measurement, we still need to measure DBP. There is no need emphasizing on the clinical relevance of SBP over DBP^35^; elevated SBP predicts the risk of cardiovascular disease better than DBP^36^.

Studies have shown that although SBP measurements are more variable, it is easier to be measured and also serve as an appropriate tool for correct risk classification than DBP. BP is associated with age^36^. Among people aged 50, hypertension is associated with risk of coronary heart disease, left ventricular hypertrophy, congestive heart failure, renal failure, and mortality than DBP^36^. Basile^36^ revealed that a stiffened arterial tree, given the same stroke volume and ejection rate, will produce a higher SBP, a lower diastolic BP (DBP) and a wider pulse pressure (PP), but the mean BP (MBP) will be unchanged, emphasizing on the importance of SBP as a determinant of cardiovascular event/outcome. Imai and colleagues^37^ examined risk factors of BP using community-based sample in northeastern Japan. They found that age, heart rate, and pulse rate are associated with BP. Several other studies have evaluated the effect of various factors on HBP or hypertension^38,39^. These authors examined factors such as geographical location or country of origin, gender and age.

Wang et al. (pp. 1555–1562) study on influences of obesity on BP in China shows that measurement such as visceral fat index (VFI) or the VFI to percentage body fat ratio (VFI/PBF) is likely to provide better understanding of adiposity-related risks for hypertension^40,41^. Meta-analysis of a randomized control trial of potassium supplementation revealed that a significant reduction in BP is associated with potassium supplementation among trial subjects who are not on any anti-hypertensive medication^40–42^. Laine et al.^43^ published work on relationship between exercise and hypertension showed that vigorous physical activity during young adulthood is associated with lower age-adjusted prevalence of hypertension compared to those who did not. Stress is also another factor found to be associated with BP elevation in both gender and elevated night-time BP among men^44^. Other factors associated with BP are foetal growth restriction or prematurity, organ damage, cardiovascular risk predictor^45^, N-terminal proB-type natriuretic peptide (NT-proBNP), where association of BP variability with NT-proBNP levels may give an indication that blood pressure variability is associated with organ damage or complications^46^.

Various authors have studied systolic BP and associated factors using different methods. Alexander et al.^47^ used the Multivariable Cox survival model to determine the effect of the burden of systolic and diastolic hypertension on a composite outcome of myocardial infarction, ischemic stroke, or hemorrhagic stroke over a period of 8 years. Their study also adjusted for the effects of demographic characteristics and coexisting conditions. In order to evaluate the association between SBP and the burden of different causes of death and disability by age and sex, Forouzanfar et al.^12,13^ used Spatiotemporal Gaussian process regression to produce mean SBP and adjusted variance for each age, sex, country, and year. Their results revealed that SBP-related deaths is associated with ischemic heart disease, hemorrhagic stroke, and ischemic stroke. In Ghana there is evidence of hypertension control benefits, however, the control rates are low^16^. Okai and colleagues^16^ assessed the associations between patient background characteristics and hypertension control using Chi-Square tests and studied the relationship between these characteristics and hypertension control using logistic regression. On the other hand, Tannor et al.^18^ used simple and multiple logistic regression determine the risk factors of hypertension in Ghana.

Taking multiple SBP/DSP measurements has proven to provide BP reading close to the true BP status of the individual^48–50^. This paper considered analysis of BP measurements taken repeatedly on the same subject within 3 minutes between each measurement, but at the same visit, using the mixed effects logistic regression model^51–53^. Taking multiple measurements provides more information and hence improves precision of estimates for appropriate policy formulations towards blood pressure control. The key hypothesis that drives this study is that high blood pressure is associated with some of the exposure variables in this study.

## Methods

In this section, we give a brief description of the data. We also discuss the outcome variable in this study as well measurement scales of risk factors used. We then discuss the methods for modeling the outcome variable. Here, we will discuss the structure of the BP variable. This provides a framework for building a model that establishes the relationship between the outcome variable and the various risk factors. Our model formulation is based on the logistic mixed effects model^54^ and we will assess the effect of gender on BP over the three BP measurements adjusting for other risk factors of BP in Ghana.

### Data and variables

The data used in this study were obtained from World Health Organization (WHO) study on Global AGEing and Adult Health (SAGE). The data collection was completed in 2014/15 and data were released in the public domain in 2020^55^. The data used in this analysis consist of several variables including systolic and diastolic BP data on 3,809 Ghanaians in 2015.

#### Outcome variable

The outcome variable used in this study is BP. The BP measurements are categorized as high BP ($\ge$ 130/80 mmHg) or normal BP ($\le$ 130/80 mmHg). The BP variable takes a value of 1 if BP measurement is ($\ge$ 130/80 mmHg) or 0 if BP measurement is ($\le$ 130/80 mmHg). Both SBP and DBP data were collected 3 times on the same subject with one minute between each measurement for both males and females.

#### Risk factors

This study considers several risk factors of BP. However, detailed discussion and conclusions would be based on only the significant risk factors from the best fitting model. We considered continuous risk factors as weight in kg and height cm. The categorical variable considered are age (takes value of 1 if age is 60 years and above and 0 if age is less than 60 years), gender (takes value of 1 if male or 0 if female), marital status (takes the value of 0 if never married, 1 if currently married, 2 if cohabiting, 3 if divorced, or 4 if widowed), ever been to school (1 if yes or 0 if no), ever feel sad (0 if no, 1 if mild, 2 if moderate, 3 if severe or 4 if extreme), ever feel worried (0 if no, 1 if mild, 2 if moderate, 3 if severe or 4 if extreme), ever smoked tobacco (1 if yes or 0 if no), ever consumed alcohol (1 if yes or 0 if no), work involves vigorous exercise (1 if yes or 0 if no), ever diagnosed with arthritis (1 if yes or 0 if no), ever diagnosed with angina (1 if yes or 0 if no), ever diagnosed with diabetes (1 if ye or 0 if no), ever diagnosed with asthma (1 if yes or 0 if no), fruits servings (1 if fruits servings is at leas 2 cups per day or 0 if fruits servings is less than 2 cup served per day). Descriptive statistics of these variables including the outcome variable are shown in The Table 1, 2.

### Logistic mixed effects model

Since the outcome variable consist of binary repeated records on the status of BP, the logistic mixed effects model (LMM)^56^ is assumed for the BP status variable. This is because the LMM is used to model repeated measurements on the same subject by adding a subject-specific random effect to the model. This random effect captures all the unobserved subject-specific characteristics. The logistic mixed effects regression model is used to model binary outcome variables where the log odds of the outcomes are modeled as a linear combination of the risk factors when there are both fixed and random effects.

Let ${\textbf{Y}}_{i} = \left( Y_{i1}, Y_{i2}, \ldots , Y_{in} \right)$ denotes an N-dimensional vector of BP status (1 or 0) for the $i{\text {th}}$ subject. We note that the response ${Y}_{ij}$ is recorded for each subject $i, i = 1, \ldots , N$ at time $j, j = 1, \ldots , n$. In this study, all measurements were observed, there is no missing values^57–59^. The interest is how the status of the response is affected by the ${\textbf{X}}_{i}$, an $N \times p$ design matrix of risk factor for the $i^{\text {th}}$ subject. The general form of the LMM can be written as^57–59^

1 $$\begin{aligned} \text {logit}\left( E\left( Y_{ij} = \pi _{ij} = 1 \mid X_{i}, b_i\right) \right) =\log \left( \frac{\pi _{ij}}{1-\pi _{ij}}\right) =\beta _1 X_{1 i}+\beta _2 X_{2 i}+\cdots +\beta _p X_{p i} + Z_{i}b_i, \end{aligned}$$

where $\pmb {\beta }$ is a $p \times 1$ vector of the fixed effects; which represents the effects of the risk factors on the BP, $\pi _{ij}$ is the probability of having HBP, ${\textbf{Z}}_{i}$ is a $N \times q$ design matrix for the *q* random effects, ${\textbf{b}}_{i}$ are patient-specific random effect and is assumed to follow the normal distributions such that ${\textbf{b}}_{i} \sim N \left( \pmb {0}, \sigma ^2_{b}\right)$.

From model (1), our potential logistic mixed model to be fitted can be written as

2 $$\begin{aligned} \log \left( \frac{\pi _{ij}}{1-\pi _{ij}}\right)=\,& {} \beta _{0} + \beta _{1} \times \text {Gender}_{i} + \beta _{2} \times \text {Married}_{i} + \beta _{3}\times \text {School}_{i} + \beta _{4} \times \text {Sad}_{i} + \beta _{5}\times \text {Worried}_{i} \nonumber \\{} & {} + \, \beta _{6}\times \text {Tobacco}_{i} + \beta _{7} \times \text {Alcohol}_{i} + \beta _{8}\times \text {Exe}_{i} + \beta _{9}\times \text {Arthritis}_{i} + \beta _{10}\times \text {Angina}_{i} \nonumber \\{} & {} + \, \beta _{11}\times \text {Diabetes}_{i} + \beta _{12}\times \text {Asthma}_{i} + \beta _{14}\times \text {Age}_{i} + \beta _{15}\times \text {Weight}_{i} + \beta _{16}\times \text {Weight}_{i} \nonumber \\{} & {} + \, \beta _{17}\times \text {Fruit}_{i} + \beta _{18}\times \text {Veg}_{i} + \beta _{19}\times \text {Time}_{ij} + \text {b}_{0i} + {\epsilon }_{ij}, \end{aligned}$$

where $\text {b}_{0i}$ represents the patient-specific random effect. It is assumed that $\text {b}_{0i}$ are independently distributed as $\text {b}_{0i} \sim N\left( 0,\sigma ^2_{b0}\right)$. Note that the variable for gender is **Gender**, Marital status is **Married**, ever been to school is **School**, ever feel sad is **Sad**, ever feel worried is **Worried**, ever smoke tobacco is **Tobacco**, ever consume alcohol is **Alcohol**, work involves vigorous exercise is **Exe**, ever diagnosed with arthritis is **Arthritis**, ever diagnosed with angina is **Angina**, ever diagnosed with diabetes is **Diabetes**, ever diagnosed with asthma **Asthma**, ever diagnosed with hypertension is **Hypertension**, age category is **Age**, height in cm is **Height**, weight in kg is **Weight**, fruits servings per day is **Fruit**, vegetables servings per day is **Veg** and time is **Time**.

## Analysis and results

In this section, we first present descriptive statistics of the variables in the data and then performed bivariate analysis on BP and each of the categorical risk factors using the Chi-Square test^60–62^ of association and the continuous risk factors using the t-test^63–66^. The purpose of this exercise is to identify potential risk factors of BP. Significant risk factors under these analyses were then used in the logistic mixed effects model to determine their individual contributions to BP after adjusting for other risk factors. Also, in the logistic mixed effects model, variables that were found to be statistically insignificant after adjusting for other risk factors were removed from the model. Statistical analyses in this paper were performed using R version 4.2.2.^67^. Logistic mixed effects models^52,53,68–70^ were fitted using the **glmer** function and performance of the fitted models compared and best model selected using the Akaike’s information criterion (AIC)^71–75^ and Bayesian information criterion (BIC)^75–78^.

### Descriptive statistics

Table 1, 2 shows that out of the total number of 3809 Ghanaians enrolled into the study, 1976 (51.88%) are males and 1833 (48.12) are females. Out of the 3809, 2080 (54.60%) have high blood pressure and 1729 (45.40%) have normal blood pressure (NBP). High proportion, 2102 (55.19%), of the study participants are currently married. The proportion 1666 (43.74%) of those who have never been to school lower than the proportion, 2143 (56.26%), of those who have ever been to school. The proportion, 1791 (47.02%) of the study participants who never feel sad is higher than those whose sadness levels can be described as mild followed by moderate, severe, and extreme. The proportion, 1532 (40.22%) of the study participants who never feel worried is high relative to those who extremely feel worried followed by those whose feeling is described as mild, moderate and severe. We observed that small proportion, 987 (25.94%), of the study subjects ever smokes tobacco compared with a proportion 2818 (74.06%) never smoke tobacco. Majority, 2210 (58.08%), of the study subjects ever consume alcohol and high proportion, 2146 (56.40%), of the study participants’ work does not involve vigorous exercise.

Only 524 (13.77%) of the subjects have ever been diagnosed with arthritis and few, 128 (3.36%) of participants has ever been diagnosed with angina. The proportions of those who have ever been diagnosed with diabetes and asthma are 154 (4.02%) and 144 (3.78%) respectively. We observed that 519 (13.64%) of the study subjects have ever been diagnosed with hypertension. Majority, 2243 (58.89%), of the study participants are at least 60 years old with those less than 60 years old representing 1566 (41.11%). The statistics also showed that high proportion, 2849 (74.80%), of the study participants have vegetables servings at least 2 cups per day. Those who have fruits serving at least 2 cups per day is lower, 1329 (34.89%), then those who have less than 2 cups per day. The average height of the study participants is approximately 165 cm and mean weight of 165 kg.

**Table 1:** Descriptive statistics of risk factors on n = 3,809 subjects.

Risk factor	n(%)
BP	
HBP (coded 1)	2080 (54.60)
NBP (coded 0)	1729 (45.40)
Gender	
Male (coded 1)	1,976 (51.88)
Female (coded 0)	1833 (48.12)
Marital status	
Never married (coded 0)	39 (1.02)
Currently married (coded 1)	2102 (55.19)
Cohabiting (coded 2)	28 (0.74)
Divorced (coded 3)	543 (14.26)
Widowed (coded 4)	1097 (28.80)
Ever been to school ?	
Yes (coded 1)	1666 (43.74)
No (coded 0)	2143 (56.26)
Ever feel sad ?	
None (coded 0)	1791 (47.02)
Mild (coded 1)	1202 (31.56)
Moderate (coded 2)	672 (17.64 )
Severe (coded 3)	141 (3.70)
Extreme (coded 4)	3 (0.08)
Ever feel worried?	
None (coded 0)	1532 (40.22)
Mild (coded 1)	1121 (29.43)
Moderate (coded 2)	864 (22.68)
Severe (coded 3)	287 (7.53)
Extreme (coded 4)	5 (0.13)
Have you ever smoke tobacco?	
Yes (coded 1)	987 (25.94)
No (coded 0)	2818 (74.06)
Have you ever consumed alcohol?	
Yes (coded 1)	2210 (58.08)
No (coded 0)	1595 (41.92)
Your work involves vigorous exercise?	
Yes (coded 1)	1659 (43.60)
No (coded 0)	2146 (56.40)
Have you ever been diagnosed with arthritis?	
Yes (coded 1)	524 (13.77)
No (coded 0)	3281 (86.23)

**Table 2:** Descriptive statistics of risk factors on n = 3,809 subjects continue.

Risk factor	n (%)
Have you ever been diagnosed with angina?	
Yes (coded 1)	128 (3.36)
No (coded 0)	3677 (96.64)
Have you ever been diagnosed with diabetes?	
Yes (coded 1)	153 (4.02)
No (coded 0)	3652 (95.98)
Have you ever been diagnosed with asthma?	
Yes (coded 1)	144 (3.78)
No (coded 0)	3661 (96.22)
Have you ever been diagnosed with hypertension?	
Yes (coded 1)	519 (13.64)
No (coded 0)	3286 (86.36 )
Age group	
Old (coded 1: age $\ge 60$ years)	2243 (58.89)
Young (coded 2: age $<60$ years)	1566 (41.11)
Number of times Vegetables served per day?	
$\ge 2$ cups (coded 1)	2849 (74.80)
$<2$ cups (coded 0)	960 (25.20)
Number of times fruits served per day?	
$\ge 2$ cups (coded 1)	1329 (34.89)
$<2$ cups (coded 0)	2480 (65.11)
Continuous risk factors	Mean ± standard deviation)
Height cm	165.29 ± 55.328
Weight kg	62.52 ± 45.763

### Bivariate analysis of BP versus risk factors

We carried out bivariate analysis between the BP as a binary response variable versus categorical and continuous risk factors of BP. For the categorical risk factors, we used the Chi-Square test of association to test for significance of association between the two variables (binary response versus categorical risk factors). For the continuous risk factors, we used the t-test to test for significant difference in the means of the continuous risk factors between the two groups (high blood pressure and normal blood pressure). The categorical and continuous risk factors are shown in Tables 1, 2. The Chi-Square and t-test results in Table 3 presents only risk factors that are significantly associated with the response variable (blood pressure). These significant variables are then considered in our subsequent analysis using the logistic mixed effects model (1).

### Logistic mixed effects model

Based on the significant risk factors (in Tables 1, 2), our logistic mixed effects model to be fitted can be written as

3 $$\begin{aligned} \log \left( \frac{\pi _{ij}}{1-\pi _{ij}}\right)= & \,{} \beta _{0} + \beta _{1} \times \text {Gender}_{i} + \beta _{2} \times \text {Married}_{i} + \beta _{3} \times \text {Sad}_{i} + \beta _{4}\times \text {Worried}_{ij} \nonumber \\{} & {} + \, \beta _{5}\times \text {Tobacco}_{i} + \beta _{6} \times \text {Alcohol}_{i} + \beta _{7}\times \text {Arthritis}_{i} + \beta _{8}\times \text {Diabetes}_{i} \nonumber \\{} & {} + \, \beta _{9}\times \text {Age}_{i} + \beta _{10}\times \text {Veg}_{i} + \beta _{11}\times \text {Weight}_{i} + \beta _{12}\times \text {Height}_{i} + \beta _{13}\times \text {Time}_{ij} \nonumber \\{} & {} + \, \beta _{14}\times \text {Angina}_{i} + \beta _{15}\times \text {Fruits}_{i} + \beta _{16}\times \text {School}_{i} + \text {b}_{0i} + {\epsilon }_{ij}, \end{aligned}$$

Only risk factors in the best fitting model will be discussed in detailed in the discussion and conclusion Sect. “Outcome variable”. To select the best fitting model for the BP variable, we performed step-wise variable selection procedure^79–81^.

**Table 3:** Test of association between BP versus risk factors of BP.

	Blood pressure	
Risk factor	HBP (1)	NBP (0)
Gender	Chi-Square = 65.742 , *p* value$<0.0001$	
1	1007	969
0	1073	760
Marital status?	Chi-Square = 111.078, *p* value =$<0.0001$	
0	22	17
1	1071	1031
2	11	17
3	297	246
4	678	419
Ever feel sad?	Chi-Square = 30.471, *p* value =$<0.0001$	
0	1009	782
1	630	572
2	353	319
3	85	56
4	3	0
Ever feel worried	Chi-Square = 46.059, *p* value =$<0.0001$	
0	886	646
1	584	537
2	452	412
3	153	134
4	5	0
Ever smoked tobacco?	Chi-Square = 27.063, *p* value =$<0.0001$	
1	498	489
0	1,579	1239
Have you ever consumed alcohol?	Chi-Square = 17.9018 , *p* value =$<0.0001$	
1	1169	1041
0	908	687
Ever been diagnosed with arthritis?	Chi-Square = 11.777, *p* value = 0.0010	
1	307	217
0	1,770	1511
Ever been diagnosed with diabetes?	Chi-Square = 20.616, *p* value =$<0.0001$	
1	99	54
0	1978	1674
Ever been diagnosed with hypertension?	Chi-Square = 282.926, *p* value =$<0.0001$	
1	386	133
0	1691	1595
Age	Chi-Square = 29.011, *p* value =$<0.0001$	
1	1272	971
0	808	758
Vegetables	Chi-Square = 16.004, *p* value =$<0.0001$	
1	1586	1263
0	493	467
T-test	${\bar{x}}$(95% CI)	${\bar{x}}$(95% CI)
Weight (kg)	64.119 (63.05, 65.19)	60.61 (59.27, 61.96)

Our best fitting model from the variable selection procedure is specified in Model (4) and the results are presented in Table 4.

4 $$\begin{aligned} \log \left( \frac{\pi _{ij}}{1-\pi _{ij}}\right)= & \,{} \beta _{0} + \beta _{1} \times \text {Time}_{ij} + \beta _{2} \times \text {Gender}_{i} + \beta _{3} \times \text {School}_{i} + \beta _{4} \times \text {Mild}_{i} \nonumber \\{} & {} + \, \beta _{5}\times \text {Moderate}_{i} + \beta _{6}\times \text {Severe}_{i} + \beta _{7} \times \text {Age}_{i} + \beta _{8}\times \text {Veg}_{i} + \beta _{9}\times \text {Exe}_{i} \nonumber \\{} & {} + \, \beta _{10}\times \text {Weight}_{i} + \beta _{11}\times \text {Height}_{i} + \beta _{12}\times \text {Diabetes}_{i} + \text {b}_{0i} + {\epsilon }_{ij}, \end{aligned}$$

where **Exe** is vigorous exercise, **Veg** is vegetables servings, and **Mild**, **Moderate**, and **Server** represent those who describe their sadness level as mild, moderate and severe respectively.

**Table 4:** Parameter estimates from the best fitting Model (4).

	Model (4)		
Parameter	OR	s.e	95% CI
Intercept	3.893	0.3962	(1.7908, 8.4661)
Time	0.631	0.0416	(0.5814, 0.6843)
Gender	0.274	0.2008	(0.1847, 0.4058)
Education	1.649	0.2016	(1.1108, 2.4486)
Mild	0.409	0.2270	(0.2618, 0.6375)
Moderate	0.366	0.2469	(0.2254, 0.5933)
Severe	0.382	0.3767	(0.1827, 0.8000)
Age	2.771	0.2017	(1.8658, 4.1145)
Vegetable servings	2.090	0.2154	(1.3704, 3.1888)
Vigorous exercise	1.631	0.1939	(1.1151, 2.3854)
Weight	1.009	0.0024	(1.0044, 1.0137)
Height	0.996	0.0018	(0.9921, 0.9993)
Diabetes	4.896	0.4687	(1.9535, 1.2268)

The results in Table 4 showed that the risk (OR = 0.631, 95% CI = 0.5814, 0.6843) of high blood pressure (HBP) decreases across the three measurement periods. There is significant reduction in the risk (OR = 0.2740, 95% CI = 0.1847, 0.4058) of HBP among male participants relative to female participants. We found that those who have ever felt sad (mild, moderate or severe) have reduced risk of HBP compared with those who have never felt sad. There is approximately 2-folds increase in the risk (OR = 1.649, 95% CI = 1.1108, 2.4486) of HBP among those who have formal education relative to those who has no formal eduction. The study results revealed that there is 2.77-folds increase in the risk (OR = 2.771, 95% CI = 1.8658, 4.1145) of HBP among those who are 60 years and above relative to those below the age of 60 years. The results also revealed that those who have vegetable servings at least 2 cup per day have increase risk of HBP. We observed that those whose work involves/requires vigorous exercise has 1.631-folds increase in the risk (OR = 1.631, 95% CI = 1.1151, 2.3854) of HBP relative to those whose work does not involve vigorous exercise. The risk of HBP increases with increasing weight. However, a reduced risk of HBP is associated with increasing height. Diabetes was found to be positively associated with an increase risk of high blood pressured.

## Discussion and conclusions

In this paper, we investigated the effects of variables as potential risk factors of BP. We provided summary of statistics of the variables used and then performed Chi-Square test of association to predetermine variables that may be identified as potential risk factors for inclusion in the subsequent analysis. In this study, we modeled the BP data using the mixed effects logistic regression model^56^.

Our study results revealed that SBP significantly decreases across the three measurement times. This finding suggests that there is some level of variability associated with measuring BP, even in the same subject, and hence using a single value as a measure of BP may produce BP values that are not actual representation of one’s BP level. This finding agrees with findings of various authors^48–50^ that SBP measurements within one minute between measurements is associated with decreasing SBP. Repeatedly measuring SBP provides a means of validating the accuracy of the SBP as well as the appropriate SBP of a given subject. That is studies should be design to repeatedly measure SBP on the same subject to aid in making decision on the actual BP of the subject. This finding agrees with the standard practice of using multiple SBP values to determine the SBP actual value of an individual.

Higher SBP is observed among females subjects compared to their male counterparts. This finding is contrary to the finding by Hussein and colleagues^82,83^ where SBP was found to be significantly higher in males relative to females. This is often the case when studies participants are younger age group. In Hussein and colleagues^82^ paper, ages of participants range from 18 to 26 years. In this study, ages of the study participants range from 51 to 114 years and 2199 (58.67%) of the participants are more than 50 years. Systolic BP is lower in female during early adulthood and the reverse is true for 50 years and above^83,84^. High BP related hypertension is associated with increasing age and is higher among men at younger age and higher among women at older age. The gender difference in BP control is largely determined by sex hormones in females which shows to be protective against high BP^83,84^. However, BP to increase among menopausal women since BP becomes salt-sensitive after menopause^83,84^. The implication of this finding is that females are at high risk of higher SBP at old age relative to young age. This calls for blood pressure control programs to pay special attention to both gender groups in relation to their respective age categories in which their are noted to be relatively more susceptible to high SBP. Specifically, blood pressure control programs should focus more on women at menopause since women are highly susceptible to high SBP at this stage of their life.

This study results also revealed that high BP is associated with the older age. This finding is in line with findings from various studies^82,83,85,86^. From pathophysiology perspective, high BP with increasing age is mostly related to changes in arterial and arteriolar stiffness^87^. That is, large artery stiffness is often caused by arteriosclerotic structural alterations and calcification, which the leads to earlier reflected pressure waves from the arterioles towards the heart during BP wave propagation^87^. Various studies^88–90^ showed that high BP related hypertension is associated with increasing age, where BP is lower among women at younger age and high among women at older age. The chance of having high blood pressure BP increases as you get older, especially isolated systolic hypertension. Before the age of 55 years, men are more likely to experience high BP pressure. However, after the age of 55 years, women are more likely to experience high BP. Blood pressure increase with increasing age is a major risk factor for cardiovascular and renal disease, stroke, and type 2 diabetes mellitus^89,90^ and age-related increases in blood pressure have been observed in almost every population. The China Peace Collaborative Group^91^ revealed that high blood pressure is associated with increasing age and that the risk of high BP almost 3-folds across subgroups, indicating subgroup differences in biology, behaviour, or exposures. These authors noted that anti-hypertension strongly reduces the association between age and blood pressure as well as diminishes the variation^91^. However, there some exposure groups where age and blood pressure association is negative. That is, among certain group of individuals, BP may decrease with increasing age and mostly occur among people suffering from illness such as Alzheimer’s and other forms of dementia, cancer or impaired ventricular function which may occur after myocardial infarction^87^. Michael and colleagues^89,90^ noted that increase in BP is observed in almost every population except hunter-gatherers, farmers, and pastoralists.

We found an increased risk of high BP with increasing weight. Our finding agrees other studies^82,83,85,86,92^ in the literature. These studies revealed that overweight subjects have more body fat which increases the BP than that of the normal weight subjects. Identifying factors responsible for changes in BP levels became an important topic upon which an initial group of articles focused on^40,41,93^. In both children and adults, the association between obesity and hypertension is well established. However, the pathogenesis of obesity induced hypertension is an active area of research^93^. Although it is well established that increase in the risk of hypertension is associated with obesity, it is also known that many obese individuals do not develop hypertension^40,41^. Studying the influence of interaction between air pollution and obesity on blood pressure in Chinese children in China^94^ shows that obesity increases the association of long-term air pollution with blood pressure and hypertension in Chinese. Also, investigating the influence of obesity on BP in a large population study in China by Wang and colleagues^40,41,95^ in children showed measurements of the visceral fat index (VFI) or of the VFI to percentage body fat ratio (VFI/PBF) may offer a better understanding of adiposity-related risks for hypertension and pre-hypertension. Body mass index (BMI) is stronger predictor of BP^96^. Common conclusions or recommendations among researcher is that reducing or eliminating overweight and obesity has the potential which could lead to a reduction in the risk of high BP^97^ and Bin and colleagues^98^ noted that eliminating overweight and obesity could reduce 14.4% of high BP cases among Chinese children. Even in children (both boys and girls), the prevalence of high BP is high and overweight and obese children had a significantly higher prevalence of high BP than non-overweight children^98^. There is therefore the need for primary prevention of hypertension among children and preventive measures should focus on weight control and healthy lifestyle habits. The policy implication is that BP control programs should focus on promoting weight reduction programs such as daily exercise, taking supplements/medications that burn fat, and healthy life style.

Our study results showed that there is a reduced risk of HBP among those whose work does not involve vigorous exercise compared with those whose work involves vigorous exercise. One would have expect that exercise should always decrease BP. However, BP increase or reduction is largely dependent on the degree, frequency, and duration of the exercise. A study conducted on Korean adults who self-reported that they practice regular exercise revealed that there slower rate of progression to hypertension relative to those who do not exercise^99,100^. Kim and colleagues^99^ study revealed that study participants whose level of physical activity is low are exposed to higher risk hypertension when compared with individuals whose physical activity level is moderate^100,101^. Progression to hypertension is associated with an increased risk of high BP among those whose work requires vigorous exercise. Faselis and colleagues^102^ studied male veterans in the United State of America found that there was high risk of progression to hypertension with associated decrease in cardiorespiratory fitness. Also, vigorous physical activity during young adulthood is protective against hypertension in the future. Reports on former elite athletes versus controls (those who are not former athletes) showed lower age-adjusted risk of hypertension among former elite athletes relative to the controls^103^. These authors noted that lower prevalence of hypertension is not only associated with such athletes in the future, but also the current volume of leisure-time physical activity is inversely related to the presence of hypertension^103^. For the purpose of reducing BP, individuals are recommended to engage in moderate exercise^104^. Various hypertension guidelines^105,106^ recommended aerobic exercise such as jogging, walking, and swimming for management of high BP related hypertension. Some of the guidelines include the Canada’s 2018 hypertension guidelines^107^, the Japanese Society of Hypertension Guidelines for the Management of Hypertension^108^, and 2010 Chinese guidelines for the management of hypertension^109^. WHO guidelines on management of hypertension recommend regular aerobic exercise for more than 30 min every day^110^. There is therefore the need for blood pressure control authorities to intensify campaign on need for daily exercise during young age and old age.

We found that BP is high among those who have vegetable servings at least 2 cups per day. Also, normal blood pressure is associated with those who say they have ever felt sad (mild, moderate or severe) relative to those who never felt sad. These findings suggest that using ever felt sad as a measure BP status may not be appropriate since BP level may return to normal after spike in sadness (depression or anxiety). Studies have also shown that there is no justification that anxiety and depression are directly linked to high blood pressure. This means that being sad can cause a steep rise in blood pressure. However, the blood pressure returns to its original level after the sadness. One’s feeling (anxiety or depressed) should not be used as a measure of BP status since these conditions can be sudden and may not last long with bloop pressure returning to normalcy.

The results revealed that a reduced risk of HBP is associated with increasing height; a finding which agrees with various studies in the literature^111^. A study by Stanaway and colleagues^112^ have shown that height is positively associated with hypertension. The problem with this finding is that the results was difficult to interpret. This is because the body size, which was adjusted for during the analyses, was correlated with adult height. The implication is that the body size/weight could be a mediator instead of confounder in the association between height and BP^113^. Subsequent to this finding, Bourgeois and colleagues^113^ found a negative association between height and SBP and pulse pressure as well as positive association between height and DBP. Various studies studies have established that there is an inverse relationship between height and BP^114,115^ and Das Gupta and colleagues found that for each 10cm increase in height, the odds of hypertension decreases 10% in adult Nepalese population^116^. Several factors account for this inverse association between height and BP/Hypertension^111^. For instance, an increases in height leads to a corresponding increase in the diameter of the coronary vessels^111^. These anatomical factors reduces the risk of atherosclerosis and hypertension^111,117^. Importantly, there proper lung function among taller individuals compared with short stature people and this may be responsible for the inverse relationship between height and hypertension^118–120^. Although, the mechanism explaining the relationship between height and BP/hypertension is unclear, hypertension management should centered among short individuals.

We found that there is approximately 5-fold increase in the risk of HBP among subjects who ever been diagnosed with diabetes. Our finding is in line with various studies^121^ that diabetes is a significant predictor of hypertension. Other studies^122,123^ have found that hypertension is twice as frequent in patients with diabetes compared with those who do not have diabetes. Hypertension and diabetes share common risk factors and often occur together. High blood pressure (BP) was reported as a significant predictor of type 2 diabetes^124^. It has been established that subjects with HBP have approximately 60% increased risk of developing type 2 diabetes. Various studies have identified hypertension as an independent risk factor for cardiovascular events. However, the relationship between blood pressure and the risk of new onset diabetes is remains unclear^125^.

Also, our results revealed that there is an increase risk of HBP among subjects with formal education background relative to those without formal education. Our finding is contrary to almost all the recent studies on impact of education on blood pressure and hypertension. Years of schooling was found have negative relationship with systolic blood pressure after adjusting for age, gender and race^126^. Even further adjustment for mother’s education, childhood verbal intelligence quotient, childhood health and childhood socioeconomic status was found to have little influence this conclusion. However, years of schooling became statistically insignificant (but positive association with BP) after adjusting for degree attained in the fully model^126^. Comparison of graduates versus high school degree-holders showed that graduate degree still had significantly lower systolic blood pressure than high school degree-holders^126^. As study that was designed to evaluate the impact of education on cardiovascular risk control and target BP values in hypertensive outpatients found no correlation between education intensity and the achieved BP reduction^127^. Other studies^128^ have revealed that less educated hypertensive were characterized by a significantly higher prevalence of patients with greater global cardiovascular risk rather than medium-high educated hypertensive subjects. Studies have reveled that hypertension is a prevalent condition among the least educated and poorest people in low-and middle-income countries^128,129^.

Contributions of the study; firstly, when the objective of a study is to collect data repeatedly, on the response variable, for each study participant within a specified time interval(s) or at some selected time points, then any method of analysis that assumes that such measurements in the response variable are independent is likely to produce invalid statistical inferences^54,57–59^. This means that the linear regression model that assumes that the responses are independent cannot be used to provide valid statistical inferences. In this study, we used the logistic mixed effects model^54^ to account for correlation between the repeated measurements in the BP data. The logistic mixed effects model achieves this by introducing a subject-specific random effect, which captures all unobserved subject-specific characteristics^69,70^. Secondly, this study used repeated binary BP data on 3809 Ghanaians to study the BP changes (high or low blood pressure) across the measurement periods as well determine risk factors associated with BP. In this way, one would be able to determine whether there is variability associated with BP measurements at the different periods. The study revealed that there is variability among the BP status across the measurement periods, an indication that using multiple measurements to evaluate true BP is a recommended practice and should be encourages. However, this study is unable to consider more than 3 repeated measurements to check if mean BP values would decrease or remain horizontal after some number of repeated measurements. So this study is unable to determine the number measurements required to estimate the true systolic blood pressure value in a subject under study.

## Data Availability

Supporting data for this manuscript results are available at [https://www.who.int/data/data-collection-tools/study-on-global-ageing-and-adult-health/sage-waves] upon request from “@World Health Organization (WHO) study on Global AGEing and Adult Health (SAGE)”. The authors have no right to release the data to a third party.
